# Daily Heat Stress Induces Donor‐Side Limitation of PSI Via Downregulation of the Cyt *b_6_f* Complex

**DOI:** 10.1111/pce.70730

**Published:** 2026-07-08

**Authors:** Laura Laihonen, Tutta Tomberg, Linda Vuorijoki, Lauri Nikkanen, Paula Mulo, Marjaana Rantala

**Affiliations:** ^1^ Molecular Plant Biology, Department of Life Technologies University of Turku Turku Finland

**Keywords:** acclimation, cytochrome *b*_*6*_ *f*, heat stress, photosynthesis, plants

## Abstract

The rapidly warming climate is driving increasingly frequent and intense heat waves worldwide, posing major challenges to plant metabolism and growth. Despite the central role of photosynthesis in plant growth, the effects of prolonged heat stress on the primary reactions of photosynthesis remain poorly understood. Here, we studied how long‐term heat exposure affects growth and photosynthetic performance in the model plant *Arabidopsis thaliana*. Plants were exposed daily to high temperature (38°C) for 4 h throughout the growth period. Conflicting to earlier studies, we show that PSII‐LHCII remain relatively intact under prolonged heat despite slightly reduced ∆F/F_m_’, relative electron transfer rate (rETRII) and proportion of open reaction centers (qL). Strikingly, the content of the Cytochrome *b_6_f* complex, which mediates electron transfer between the photosystems, was reduced by 30–40%. This leads to more oxidized downstream electron acceptors and prevents PSI over‐reduction in conditions where carbon assimilation is inhibited.

## Summary statement

Prolonged moderate heat does not damage PSII‐LHCII. Reduced photosynthetic efficiency is due to down‐regulated electron flow from Cyt *b*_*6*_ *f* to PSI, which protects PSI against over‐reduction in conditions where downstream metabolism is inhibited.

## 1 Introduction

The global mean temperature in 2024 was 1.5°C above the pre‐industrial era (World Meteorological Organization 2025). Global warming has led to more frequent and severe heatwaves, disrupting ecosystems and threatening agriculture. Based on different modeling and field‐warming experiments, it was estimated that each degree‐Celsius increase in global mean temperature would reduce global yields of wheat by 6.0%, rice by 3.2%, maize by 7.4%, and soybean by 3.1% (Zhao et al. 2017). At the same time, the agricultural production needs to double during this century to meet the demand of growing population. The rapidly warming climate, particularly the increased frequency and intensity of heatwaves (Intergovernmental Panel on Climate Change IPCC 2023), significantly impacts plant metabolism. Understanding the mechanisms of heat damage and acclimation are fundamental for developing mitigation strategies that prevent the loss of plant productivity under increasing temperatures.

Photosynthesis is considered to be among the most prone cell functions to be damaged upon heat stress (Allakhverdiev et al. 2008). Light‐driven photosynthetic electron transfer is conducted by membrane‐embedded large protein complexes. Photosystem (PS) II and PSI contain peripheral light harvesting antennae (LHCII and LHCI, respectively), which are interconnected by cytochrome (Cyt) *b*_*6*_ *f* and two mobile electron carriers: plastoquinone (PQ) and plastocyanin (PC). The electrons, originating from water, are transferred from one complex to another and ultimately used for reducing NADP^+^ to NADPH. Water oxidation by PSII and PQH_2_ oxidation by Cyt *b*_*6*_ *f* releases protons in the lumen. The formed proton gradient across the thylakoid membrane is released by ATP synthase with a concomitant formation of ATP. Generated NADPH and ATP are used in downstream metabolism, most importantly to assimilate atmospheric CO_2_ in the Calvin‐Benson‐Bassham (CBB) cycle. Balancing the energy supply from electron transfer reactions with the downstream metabolic demands is essential under fluctuating environmental conditions. To cope with environmental fluctuations, plants have evolved various short‐term mechanisms to regulate their photosynthetic apparatus according to environmental cues. These mechanisms involve adjustments in the light harvesting antennae to balance the amount of excitation energy between PSII and PSI (state transitions), dissipation of excess absorbed energy as heat (non‐photochemical quenching (NPQ)) and regulation of the electron transfer chain at Cyt *b*_*6*_ *f* and PSI (photosynthetic control and cyclic electron transfer (CET)). Acclimation to more long‐term changes in environmental conditions usually require stochiometric readjustments of the electron chain components.

Excessive heat affects all aspects of plant photosynthesis from electron transport to carbon metabolism (Berry and Bjorkman 1980; Inoué et al. 1987; Sharkey 2005; Sharkey and Zhang 2010). Carbon assimilation in particular is reduced due to impaired activity of Rubisco activase (RCA) (Feller et al. 1998; Law and Crafts‐Brandner 1999; Crafts‐Brandner and Salvucci 2000; Rokka et al. 2001), increased photorespiration and decreased stomatal conductance (e.g. Moore et al. 2021). The effects of high temperature stress on photosynthetic light reactions are less understood. The reported effects of high temperature on light reactions include dissociation of the peripheral LHCII antenna from PSII core (Armond et al. 1980; Sundby et al. 1986; Srivastava et al. 1997; Mathur et al. 2011), destacking of thylakoid membranes (Gounaris et al. 1983, 1984), more oxidized PQ‐pool (Pshybytko et al. 2008) and impairment of oxygen evolving complex (OEC) (Santarius 1976; Enami et al. 1994). Indeed, PSII has long been considered to be the most prone ETC component to high temperature stress (Berry and Bjorkman 1980), in comparison with the thermostable PSI (Ivanov et al. 2017). Notably, many of the existing studies are based on acute single exposure *in vitro* experiments (Gounaris et al. 1983, 1984; Sundby et al. 1986; Inoué et al. 1987; Enami et al. 1994; Semenova 2004; Komayama et al. 2007), while understanding of how long‐term, heat exposure, such as prolonged heatwave, impacts photosynthesis *in vivo* remains limited.

Here, we studied the effects of physiologically relevant long‐term heat stress on plant growth and photosynthetic reactions. *Arabidopsis thaliana* plants were subjected daily to 38°C for 4 h at mid‐day during their whole life cycle. In this setup, plants begin to acclimate to long‐term recurring elevation in temperature, but also experience short‐term stress due to daily heat periods. The effects of daily heat exposure on plant growth, carbon assimilation, electron transfer reactions and photosynthetic proteins and protein complexes were studied using chlorophyll‐fluorescence and absorbance‐based methods, immunoprobing and native‐PAGE. Consistent with earlier studies, heat‐acclimated plants showed significantly reduced carbon assimilation rates. However, in contrast to earlier work, that has primarily focused on single exposure to extreme temperatures, our results indicate that under prolonged heat stress PSII‐LHCII remain relatively intact with only modest decline with efficiency. Strikingly, Cyt *b*_*6*_ *f* is dramatically downregulated and thus limits electron supply to PSI. We propose that plants acclimate to long‐term heat stress by adjusting the stoichiometry of ETC components. The restricted electron flow from PSII functions to protect PSI complex from overreduction, with a concomitant reduction of photosynthetic activity.

## 2 Materials and Methods

### 2.1 Plant Growth Conditions and Phenotyping

*Arabidopsis thaliana* ecotype Columbia‐0 (Wt) was grown under 12 h light/12 h dark cycle at photosynthetic photon flux density (PPFD) of 130 μmol m^‐2^ s^‐1^ (VALOYA LUMI‐CS LL120 LED), + 23°C and relative humidity of 50% for 4–5 weeks. Heat‐acclimated plants were grown in a chamber where temperature elevated daily from 23°C to 38°C for 4 h and RH was 50%–70% depending on the temperature. Light intensity and cycle were same as in Ctrl.

The phenotypes of heat‐acclimated and Ctrl plants were imaged weekly throughout the growth period. The images were processed with Adobe Photoshop (version 26.6.1, Adobe Systems Inc.).

### 2.2 Pigment Analysis (DMF)

Leaf disks (63.6 mm^2^) collected from 5‐week‐old Wt plants grown under daily heat stress and Ctrl conditions were immersed in *N,N*‐dimethylformamide (DMF), then incubated overnight in darkness. Leaf debris was pelleted by centrifugation (5 min, 20 000 x g, RT), and absorbances of the supernatants were measured at 663.8, 646.8, and 480 nm with spectrophotometer (UV‐1800, Shimadzu). Pigment concentrations were calculated according to Wellburn (1994) using the following equations: Chl *a* = 12A663.8–3.11A646.8, Chl *b* = 20.78A646.8–4.88A663.8, Car = (1000A480–1.12Chl *a*–34.07Chl *b*)/245. Nine leaf disks from three biological replicates were measured (*n* = 27).

### 2.3 Transmission Electron Microscopy (TEM)

Leaf samples for TEM were prepared according to Hyman and Jarvis (2011). Briefly, the leaf fragments (1 × 5 mm^2^) from 5‐week‐old wild‐type *Arabidopsis thaliana* plants were cut at 90° angle to the midrib from leaves of same developmental stage. The samples were collected in the middle of light and heat period. Ten leaf fragments from three individual plants were dissected in fixative solution (2.5% (wt/vol) glutaraldehyde, 0.1 M sodium phosphate buffer, 4.0% (wt/vol) paraformaldehyde, pH 7.2) and incubated for 4 h at room temperature. Samples were then transferred to 4°C and stored overnight. The sample preparation for TEM was done according to Hyman and Jarvis (2011). Samples were analyzed with JEM‐1400 Plus Transmission Electron Microscope.

### 2.4 Thylakoid Isolation

Thylakoid proteins were isolated according to Järvi et al. 2011. Chl concentration was determined from the samples according to Porra et al. 1989. Isolations were done at the middle of heat period.

### 2.5 Polyacrylamide Gel Electrophoresis (PAGE)

For SDS‐PAGE, thylakoid proteins were solubilized with Laemmli buffer (Laemmli 1970) supplemented with 6 M urea and 10% β‐mercaptoethanol and run on 12% acrylamide gels. The proteins were electroblotted onto Immobilon‐FL membrane (Merck Millipore) with Trans‐blot (Bio‐Rad) device, blocked with 5% milk or bovine serum albumin (BSA) in TTBS (20 mM Tris–HCl, pH 7.5, 150 mM NaCl, and 0.05% Tween 20) and incubated with primary antibody overnight. The sample was loaded as follows: 0.5 μg of Chl for Phospho‐Thr/Ser antibody (Cell Signaling Technology, 9381S; 1:6000) and 1 μg of Chl/10 μg of protein for D1 (DE‐loop antibody (Kettunen et al. 1996); 1:8000), PsbO‐1 (AS142824, 1:5000), PSAB (AS10695; 1:2500, Agrisera), ATPB (AS05085; 1:5000, Agrisera), Cyt f (AS06119; 1:1000, Agrisera), Rieske (AS08330; 1:5000), Lhcb1 (AS01004; 1:2000), Rubisco L (AS03037, 1:5000), Rubisco activase (gift from T.J. Andrews, Rokka et al. 2001, 1:1000), and 2 µg of Chl for NdhL (1:5000) and PGR5 (1:1000) (gift from Shikanai, Shimizu et al. 2008). Samples for most of the SDS–PAGE analyzes were normalized to chlorophyll content, as this approach is commonly used to account for differences in thylakoid membrane protein abundance. Since heat grown plants exhibited lower chlorophyll content, we repeated subset of analyzes with protein‐based loading, and yielded broadly comparable results (Supporting Information Figure S1). The membrane was incubated with a secondary antibody IRDye 800CW Goat anti‐Rabbit IgG (1:20 000 in 1% milk/TTBS) and scanned with Li‐Cor Odyssey CLx.

For BN‐PAGE, gels and samples were prepared as in Rantala et al. 2018. Thylakoid samples were solubilized with 1% digitonin or β‐DMM, and samples containing 5 µg of Chl were separated on BN gels.

### 2.6 Chlorophyll a Fluorescence and P700 Absorbance Spectroscopy

DKN spectrophotometer (Heinz Walz GmbH) was used to measure Chl *a* fluorescence from PSII with pulse‐modulated 540 nm measuring light, and redox changes of P700, PC and Fd were measured by monitoring absorbance changes at 780–820, 820–870, 840–965, and 870–965 nm. PC, P700 and Fd redox changes were deconvoluted based on differential model plots (DMPs) (Klughammer and Schreiber 2016) measured specifically for Arabidopsis leaves. Normalization of PC, P700 and Fd redox changes was done according to the maximal redox changes that were determined with the software's NIRMAX script. Steady state oxidation and reduction levels were calculated as relative PC_ox_, P700_ox_ and Fd_red_. Light response curves were measured by illuminating leaves with 635 nm red actinic light for 5 min with the following light intensities: 100, 200, 500, 750. and 1200 μmol m^−2^ s^−1^. Plants were dark‐adapted for 1 h prior measurement, and measurements on heat‐acclimated plants were done in the middle of heat treatment period. Minimal and maximum fluorescence (F_o_ and F_m_) and the maximum P700 signal were determined in the dark‐adapted state. The effective PSII quantum yield of illuminated samples (∆F/F_m_’) was calculated as (F_m_’ – F)/F_m_’ (Genty et al. 1989), the fraction of open PS II centers (qL) was calculated as qL = (F_m_’ – F) × F_o_/((F_m_ – F_o_)/F_m_ + (F_o_/F_m_^’^))/(F_m_’ – (F_o_/((F_m_ – F_o_)/F_m_ + (F_o_/F_m_^’^)) × F (Oxborough and Baker 1997; Kramer et al. 2004), relative electron transfer rate of PSII as ETR (II) = (∆F/F_m_’) × PPFD × 0.84 × 0.5 (Genty et al. 1989), and non‐photochemical quenching as NPQ = (F_m_ – F_m’_)/F_m_’ (Demmig‐Adams 1990).

### 2.7 77 K Measurement

77 K fluorescence measurements were performed on thylakoids diluted in BTH buffer (25 mM BisTris/HCl (pH 7.0), 20% (wt/vol) glycerol and 0.25 mg·ml^1^ Pefabloc) to a chlorophyll concentration of 5 µg/ml. Thylakoids kept in liquid nitrogen and were excited with blue (440 nm) light and fluorescence emission was detected with QEPro spectrophotometer (Ocean Optics). Spectra were normalized to 685 nm peak.

### 2.8 ECS Measurements

The ECS signal arising from light‐induced shifts in the absorbance spectra of thylakoid pigments was measured by simultaneously monitoring the absorbance differences at 546 nm and 520 nm with a JTS‐150 pump‐probe spectrophotometer (BioLogic). The detectors were shielded form scattering effects by BG‐39 filters (Schott). Actinic light and single‐turnover flashes were provided by a 660 nm 10 W laser diode (AeroDiode). The ECS signal was deconvoluted from scattering artefacts as (A_520 nm_ x 1.611) ‐ (A_546 nm_ x 1.247) according to the scaling factors determined by Kramer and Sacksteder (1998). ECS_T_, light‐induced ECS amplitude corresponding to the magnitude of light‐induced *pmf*, was calculated as the difference between ECS in light (130 μmol m^−2^ s^−1^) and an Y_0_ value obtained from the first‐order exponential fit to the decay kinetics of the ECS signal during 350 ms dark intervals. Dark intervals were given every 15 s. The ECS signal was normalized to the ΔECS induced by a 20 µs saturating single‐turnover flash administered prior to the onset of actinic illumination. The gH^+^ parameter (thylakoid membrane conductivity to protons) was calculated as the inverse of the time constant of the first‐order exponential fit to ECS decay kinetics during a dark interval (Avenson et al. 2005; Cruz et al. 2005). The vH^+^ parameter (the rate of proton flux over the thylakoid membrane) corresponding to the initial slope of the decay of the ECS signal upon cessation of illumination was calculated as *pmf* x gH^+^ (Cruz et al. 2005). Partitioning of *pmf* between its component parts ΔpH and ΔΨ was determined by monitoring the post‐illumination kinetics of the ECS signal, and by quantifying the magnitude of the rapid decay of the signal below the dark baseline, caused by efflux of protons through ATP synthase, and the gradual increase in the signal in the dark caused by influx of counter‐ions (Cruz et al. 2005). Plants were dark‐adapted for 1 h prior measurement and measurements on heat‐acclimated plants were done both at the middle of heat treatment period and before heat treatment period.

### 2.9 Cytochrome *f* Redox Change Measurements

Cytochrome *f* redox kinetics were measured as time‐resolved absorbance changes at 554 nm with a 546 and 573 nm baseline (ΔA_554 nm_ – ((ΔA_546 nm_ + ΔA_573 nm_)/2) during the same light regime used for the ECS measurements, with the JTS‐150 spectrophotometer (BioLogic). BG39 filters (Schott) were used to shield the detectors from scattering effects. Cytochrome *f* re‐reduction rates were determined by fitting the post‐illumination decay kinetics of the signal to a first‐order exponential function y = y_0_ + A_1_ × exp(‐(x – x_0_)/t_1_), with *k*_Cyt *f* red_ = 1/t_1_. Maximal oxidation of Cyt *f*, determined by the initial decrease in the deconvoluted signal upon illumination, was used to normalize the Cyt *f* oxidation values.

### 2.10 Gas Exchange Measurements

Carbon assimilation measurements were performed using 5‐week‐old plants grown at a PPFD of 130 µmol m^−2^ s^−1^ with the GFS‐3000 gas exchange system (Walz) equipped with 3,010 M standard measuring head and 3041 L LED light source. Measurements were performed on 6 plants/condition at a humidity of 14,000 ppm, at CO_2_ concentration of 400 ppm, and the cuvette temperature was set to 20°C. CO_2_ assimilation and H_2_O evaporation were monitored at three different light intensities (200, 600, and 2000 µmol m^−2^ s^−1^) for 5 min/intensity.

## 3 Results

### 3.1 Long‐Term Daily Heat Exposure Results in Stunted Growth and Reduced Content of Photosynthetic Pigments

To study the effects of daily heat exposures on photosynthesis, plants were grown in chamber where temperature was elevated daily for 4 h from 23°C to 38°C throughout their lifecycle. Control (Ctrl) plants were grown at constant 23°C. The plants were photographed weekly from week one to five (Figure 1a) and the biophysical and biochemical characterization was conducted on plants at the end of the growth period, at week five. As demonstrated in Figure 1a (lower panel), daily heat treatment resulted in stunted growth, fresh weight of the plants reaching only ~40% of that of Ctrl plants (Figure 1a upper panel, Figure 1b), as well as altered rosette morphology with elongated petioles and leaves elevated from the soil. Additionally, strong hyponastic leaf movement was observed during the heat exposures: the petioles started to bend upwards from the soil during the high temperature period (Supporting video). Further, in heat‐acclimated plants, the accumulation of chlorophyll (Chl) *a* and *b* was reduced by ~30% with no significant effects on Chl *a/b* ratio, as compared to Ctrl (Figure 1c,d). Moreover, the accumulation of carotenoids (xantophylls and carotenes) was reduced by ~30% (Supporting Information Figure S2).

**Figure 1:**
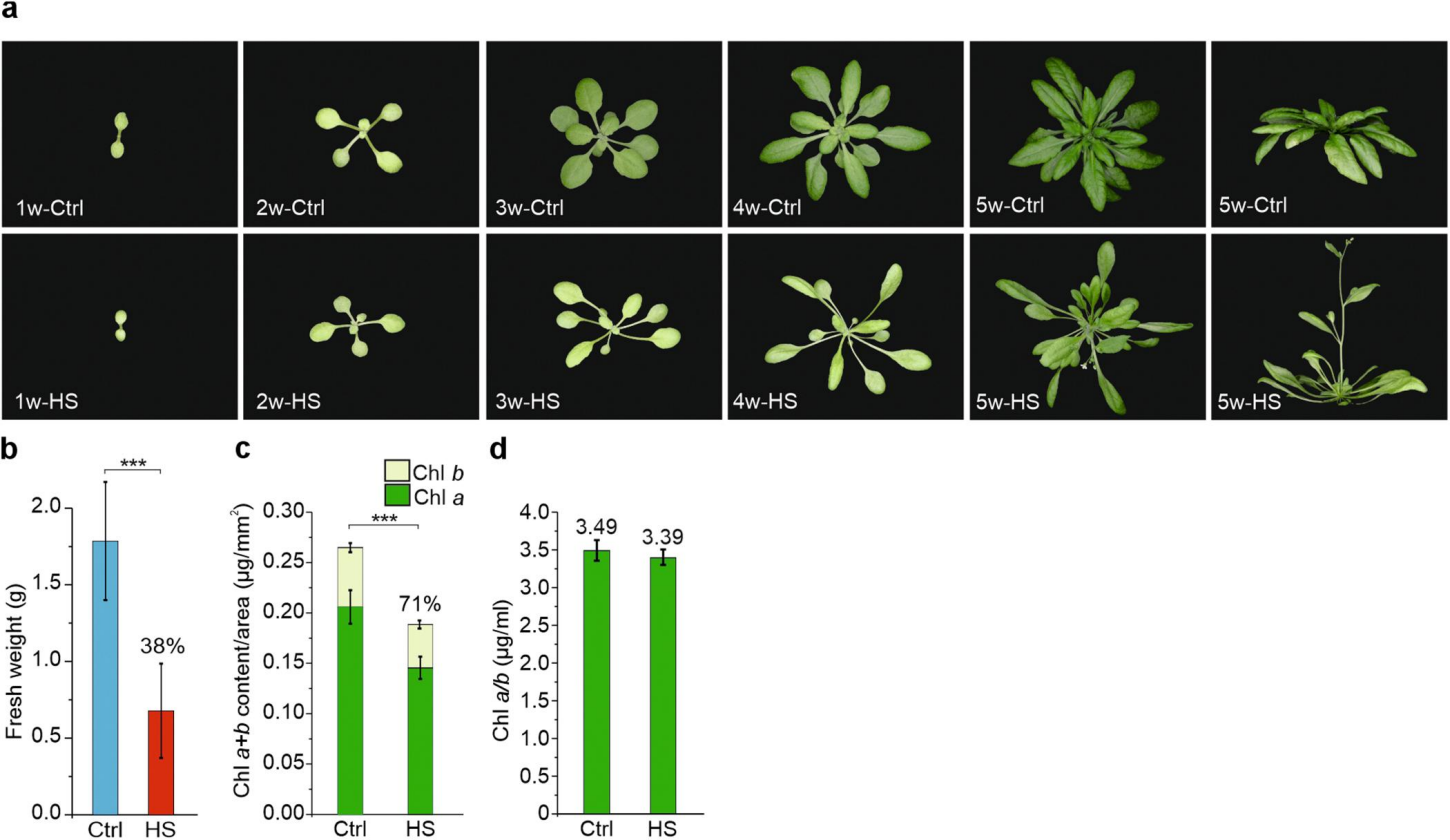
The effect of long‐term daily heat exposure on (a) plant growth phenotype, (b) fresh weight (c) chlorophyll content and (d) chlorophyll a/b ratio. Plants were grown for 5 weeks in growth chamber where temperature increased daily from 23°C to 38°C for 4 h (HS), whereas control (Ctrl) plants were grown at constant 23°C. Fresh weight (*n* = 15) and chlorophyll (Chl) content (*n* = 27) were measured at week five. Statistical significance according to Student's t‐test is indicated with an asterisk (* = < 0.05, ** = < 0.01, *** = < 0.001). Standard deviations are indicated with error bars.

### 3.2 Daily Heat Stress Alters Thylakoid Ultrastructure and Number of Grana Per Chloroplast

Earlier reports have demonstrated complete linearization of the thylakoid membrane system upon sudden exposure to high temperature (Gounaris et al. 1983, 1984). Transmission electron microscopy was used to study the effects of daily heat exposure on the thylakoid membrane ultrastructure. Leaf samples were collected from 5‐week‐old control plants and from plants grown under long‐term heat stress in the middle of the heat period. Heat‐acclimated plants showed higher number of grana per chloroplast (52 ± 2 SD) compared to Ctrl conditions (33 ± 10 SD), but the average height of grana was significantly smaller in heat‐acclimated plants (108.5 ± 61 nm SD) compared to Ctrl plants (120.4 ± 60 nm SD) (Figure 2c). Additionally, daily heat exposure resulted in larger plastoglobuli as compared to Ctrl conditions (Figure 2a,b).

**Figure 2:**
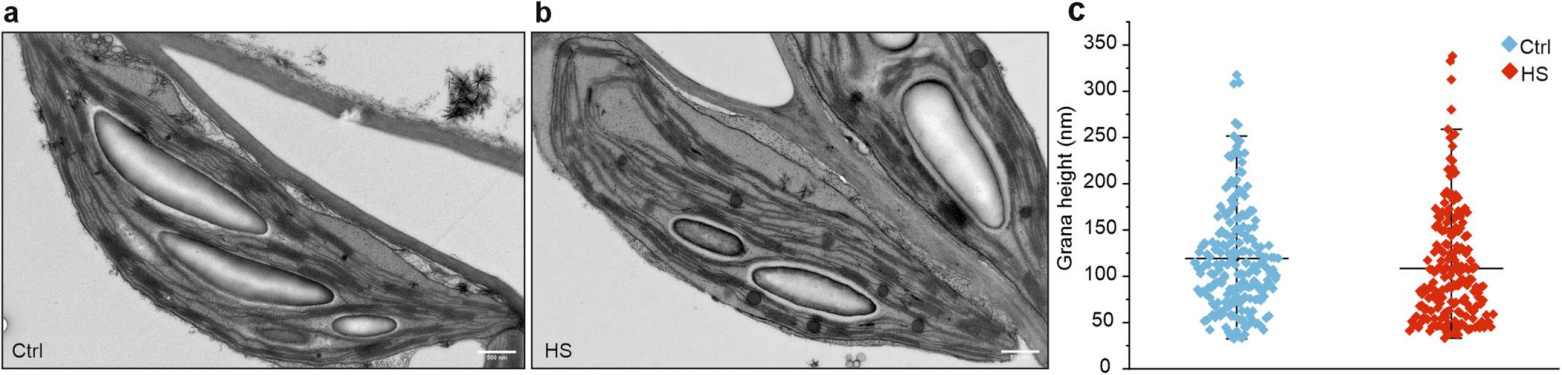
**Transmission electron microscopy analysis of chloroplast ultrastructure.** a, b) Representative electron micrographs of chloroplasts from control (Ctrl) and heat‐acclimated (HS) plants. 8000 × magnification was used; 500 nm scale bar is indicated at bottom‐right corner. c) Height of individual grana stacks was measured from 202 (Ctrl) and 194 (HS) grana stacks. Samples were collected in the middle of light and heat period. Mann‐Whitney Rank Sum Test showed statistical significance between Ctrl and HS plant grana height (*p* = 0.021). Standard deviations are indicated with error bars.

### 3.3 PSII and PSI Remain Structurally Intact but Their Photochemistry is Reduced Under Prolonged Heat Stress

Previous studies have demonstrated that PSII‐LHCII complex is the most prone ETC component to high temperature‐induced damage. Heat stress has been shown to result in LHCII disconnection from PSII (Armond et al. 1980, 1978) and degradation of the PSII core protein D1 as well as disassembly of the OEC complex (Enami et al. 1994; Yamashita et al. 2008). To investigate the effects of daily moderate heat exposures on PSII‐LHCII supercomplexes, thylakoid membranes from heat‐acclimated and Ctrl plants were isolated at mid‐day in the middle of heat period, and solubilized with non‐ionic detergent β‐dodecyl maltoside (β‐DDM), followed by separation with Blue Native (BN)‐PAGE. In contrast to previous studies, heat‐acclimated plants exhibited a stable association of LHCII with the PSII core, as evidenced by the high accumulation of large PSII‐LHCII supercomplexes, specifically C2S2M(2). These complexes consist of a PSII core dimer (C2) bound to two strongly (S2) and one or two moderately (M or M2) attached LHCII trimers (Figure 3a). Correspondingly, heat‐acclimated plants accumulated fewer C2S, C2S complexes, and detached M‐LHCII complexes compared to Ctrl plants. Additionally, heat‐acclimated plants displayed a reduced accumulation of PSII monomers (Figure 3a). However, no differences were observed in the overall abundance of the PSII reaction center protein D1 or the LHCII protein Lhcb1 between heat‐acclimated and Ctrl plants (Figure 3b). Notably, the PSII core protein CP43 as well as LHCII proteins were less phosphorylated in heat‐acclimated plants than in Ctrl (Figure 3b).

**Figure 3:**
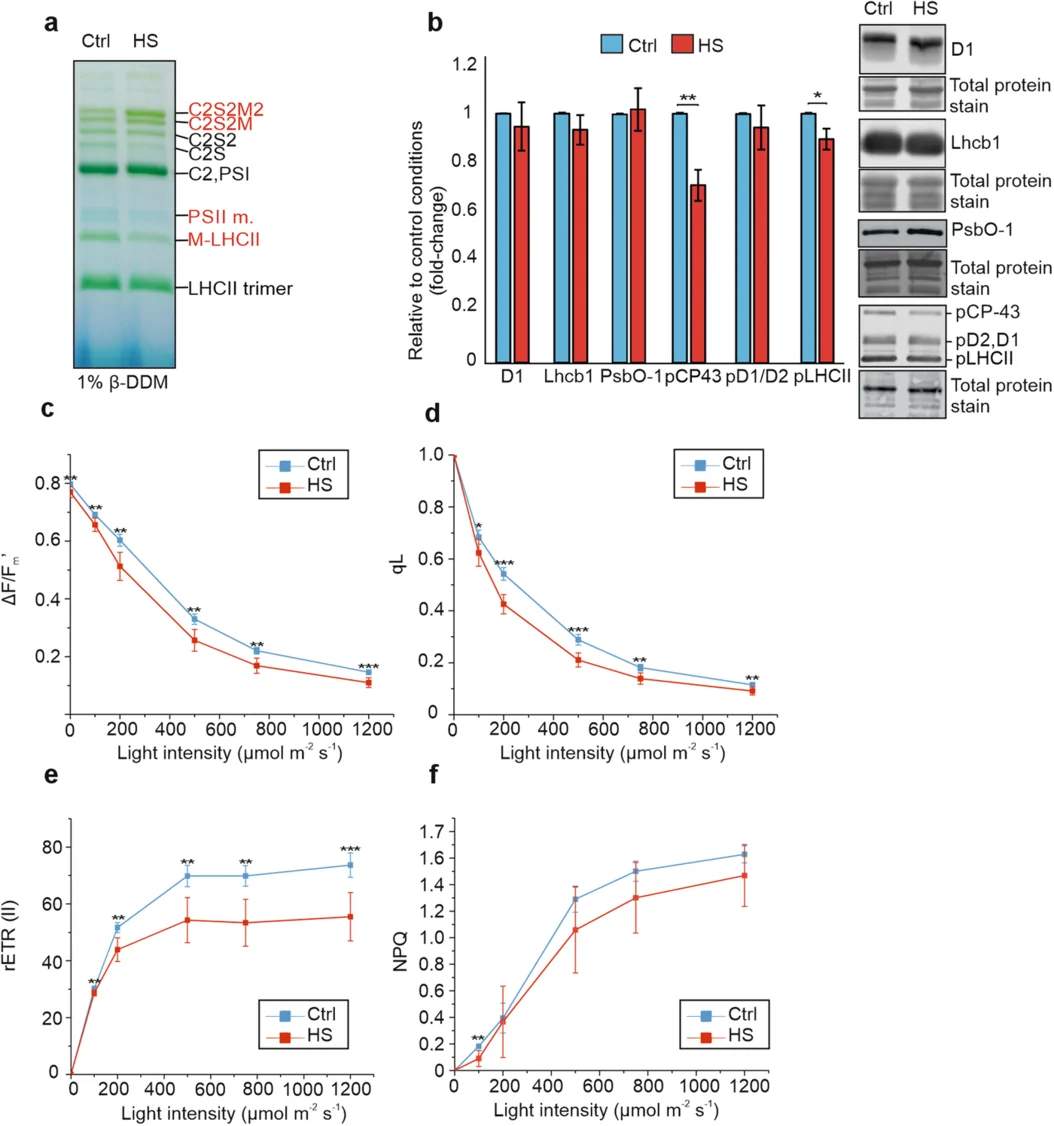
The effects of daily heat stress (HS) on PSII‐LHCII complex. a) Representative BN gel for thylakoids isolated from control (Ctrl) and HS plants using β‐dodecyl maltoside (β‐DDM) for solubilization. 5 µg of Chl was loaded per lane. Complexes showing differences between Ctrl and HS are marked in red. b) Western blots and protein quantification from thylakoid membrane proteins isolated from Ctrl and HS plants using antibodies against D1, Lhcb1 and Phosphorylated Thr/Ser. Quantifications are normalized to Ctrl. (*n* = 3 biological replicates) The membranes were stained with total protein stain to verify equal loading. c–f) Rapid light response curves were recorded using chlorophyll *a* fluorescence with Dual KLAS‐NIR system. ∆F/F_m_', proportion of open PSII reaction centers (qL), relative PSII electron transport rate (rETR(II)) and non‐photochemical quenching (NPQ) were measured. Statistical significance according to Student's t‐test is indicated with an asterisk (* = < 0.05, ** = < 0.01, *** = < 0.001). (*n* = 6 individual plants). Experiments (thylakoid membrane isolation and DKN measurements) were done at mid‐day in the middle of heat period. Standard deviations are indicated with error bars.

Despite accumulating structurally intact C2S2M2 supercomplexes, heat‐acclimated plants showed higher initial fluorescence (F_o_) and reduced maximal fluorescence (F_m_) in chlorophyll *a* fluorescence measurement (Supporting Information Data S1). Elevated F_o_ under heat stress has been reported previously (e.g., Havaux 1993; Pospíšil et al. 1998) and suggests a blockage of PSII. Havaux (1993) proposed that the PSII donor side is affected already at 32°C, whereas inhibition of electron transfer from Q_A_ to Q_B_ occurs only at 42°C or higher temperatures. However, at the protein level, we did not observe differences in the accumulation of the OEC subunit PsbO (Figure 3b). Chlorophyll *a* fluorescence measurement in the light‐adapted state showed significantly lower ΔF/F_m_′ (in which F_m_′ indicates fluorescence during light saturating point and F indicates fluorescence before the saturating flash), relative electron transport rate through PSII (rETR(II)), and the proportion of open PSII reaction centers (qL) in heat‐acclimated plants compared to controls at all measured light intensities, with high light showing more pronounced effect (Figure 3c–e). NPQ was not markedly affected in heat‐acclimated plants (Figure 3f).

PSI is considered to be relatively stable under acute heat stress (Ivanov et al. 2017), and previous studies have demonstrated enhanced LHCII phosphorylation and increased absorbance cross‐section of PSI under heat stress in darkness (Ivanov and Velitchkova 1990; Mohanty et al. 2002; Nellaepalli et al. 2011). To study PSI composition, thylakoid membranes isolated from heat‐acclimated and Ctrl plants at mid‐day, in the middle of heat period, were solubilized with digitonin in ACA buffer. In BN gels (Figure 4a), no differences were observed in the amount of PSI complex, but Western blot analysis indicated higher abundance (~20%) of PSI reaction center subunit PsaB in the heat‐acclimated plants (Figure 4b). Although there were no defects in the PSI accumulation, PSI quantum yield (Y(I)) and relative electron flow through PSI (rETR(I)) were lower in heat‐acclimated plants at nearly all light intensities except at the lowest measured light intensity (Figure 4c,d). Moreover, in line with the reduced LHCII phosphorylation level (Figure 3b), heat‐acclimated plants exhibited slightly lower accumulation of the state transition ‐specific PSI‐LHCII (Figure 4a). To study the relative excitation of PSII and PSI, low temperature fluorescence spectra was recorded. The 77 K fluorescence spectra showed slightly higher PSI excitation relative to PSII in heat‐acclimated plants, but the difference was not statistically significant (Figure 4e).

**Figure 4:**
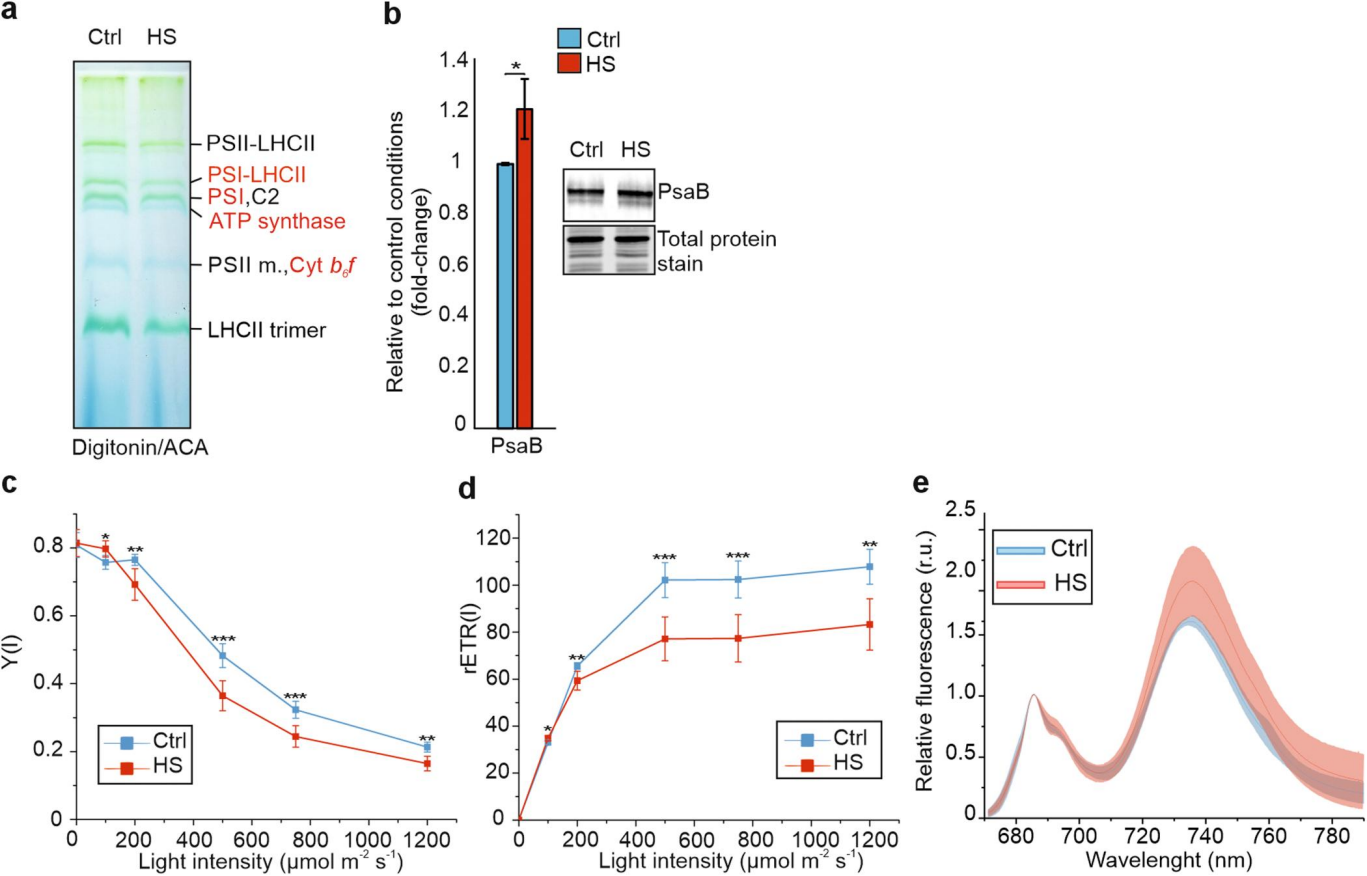
The effects of daily heat stress (HS) on photosystem PSI. a) BN‐PAGE analysis of native protein complexes from thylakoids of control (Ctrl) and heat acclimated (HS) plants using digitonin in ACA buffer for solubilization. 5 µg of Chl was loaded per lane. Complexes showing differences between Ctrl and HS are marked in red. b) Western blot analysis and quantification of PsaB protein from thylakoids isolated from Ctrl and HS. The quantifications were normalized to Ctrl. (*n* = 3 biological replicates). c and d) Rapid light response curves were recorded using P700 absorption spectroscopy with Dual KLAS‐NIR system to record. PSI quantum yield (Y(I)) and relative PSI electron transport rate (rETR(I)) were recorded (*n* = 6 individual plants). e) 77 K fluorescence spectra from Ctrl and HS thylakoids (*n* = 3 biological replicates). Statistical significance according to Student's t‐test is indicated with an asterisk (* = < 0.05, ** = < 0.01, *** = < 0.001). Experiments (thylakoid membrane isolation and DKN measurements) were done at mid‐day in the middle of heat period. Standard deviations are indicated with error bars.

### 3.4 Downregulation of Cyt b_6_f Complex Limits Electron Transfer to PSI Donor Side

To determine whether the declined PSI photochemistry detected upon daily heat exposures (Figure 4) resulted from defects in PSI donor or acceptor side, we measured the acceptor and donor side limitations of PSI and the relative redox states of P700, PC and Fd in the middle of heat period (Figure 5a–c). Figure 5a shows that Y(NA) across all light intensities is lower in heat‐acclimated plants than in Ctrl, indicating no limitations at PSI acceptor side. This result is in line with the less reduced Fd in heat‐acclimated plants as compared to Ctrl plants (Figure 5c). In contrast, donor‐side limitation, Y(ND), was substantially increased in heat‐acclimated plants (Figure 5b) and both PC and P700 were more oxidized (Figure 5c), implying that the electron flow from Cyt *b*_*6*_ *f* to PSI is restricted. This is indeed supported by the dramatically reduced Cyt *b*_*6*_ *f* content in heat‐acclimated plants, as shown by BN‐PAGE (Figure 4a). This result was confirmed by 30‐40% decrease in the content of CytF and Rieske subunits of the Cyt *b*_*6*_ *f* complex in Western blot analysis (Figure 5d). Also, the ATP synthase complex (Figure 4a) and the ATP synthase subunit ATPβ (Figure 5d) were significantly less abundant in heat‐acclimated plants as compared to the Ctrl.

**Figure 5:**
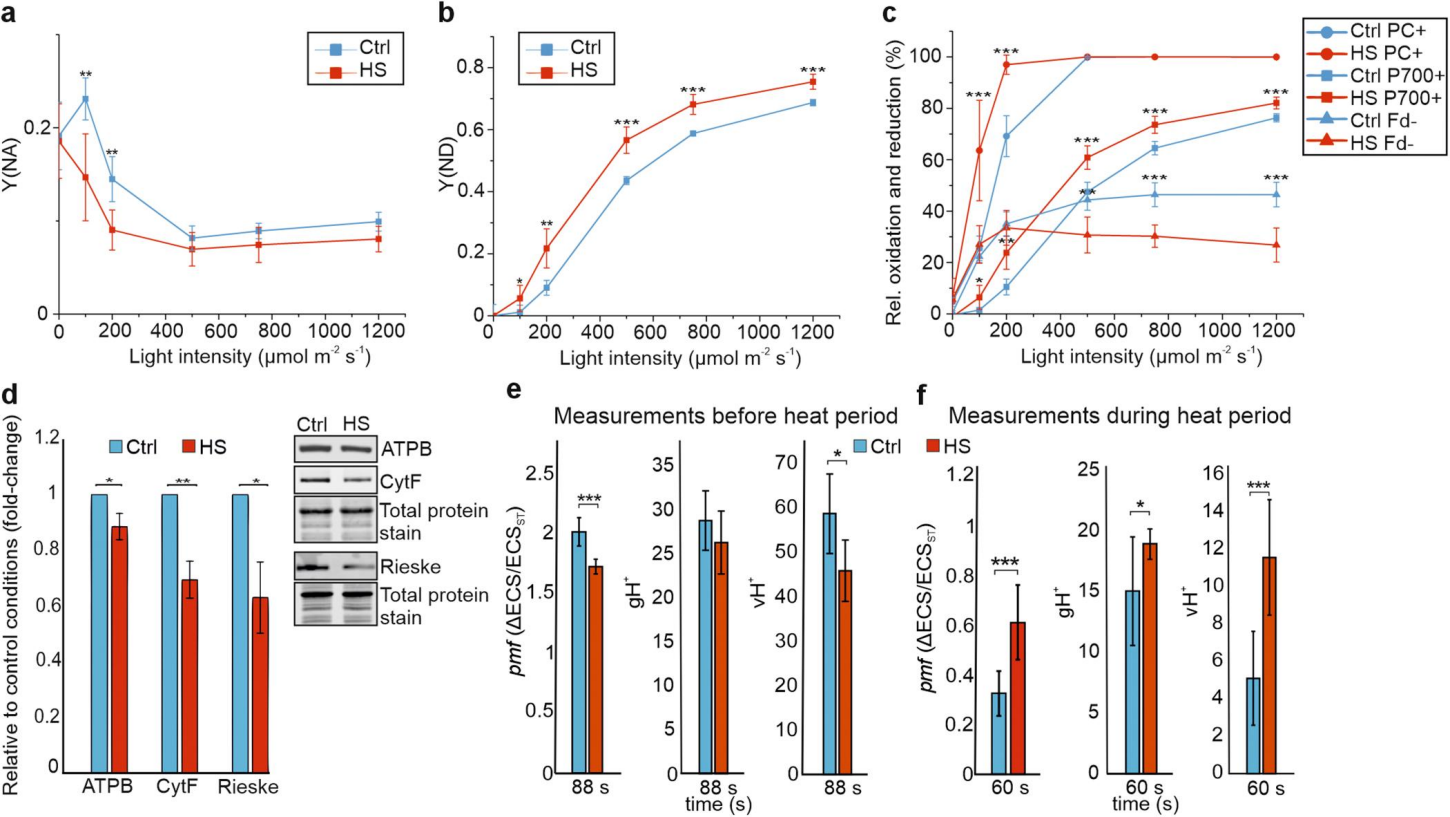
Heat stress (HS) effects on donor and acceptor side limitation of PSI and proton motive force (*pmf*). a–c) Dual KLAS‐NIR was used to measure PSI acceptor side limitation (Y(NA), PSI donor side limitation (Y(ND) and relative oxidation/reduction of plastocyanin (PC), P700 and ferredoxin (Fd). (*n* = 6 individual plants). d) Western blots and protein quantifications from thylakoids of control (Ctrl) and HS plants using antibodies against ATPB, Cyt F and Rieske. (*n* = 3 biological replicates). Thylakoid membrane isolation and DKN measurements were conducted at mid‐day in the middle of heat period. e–f) ECS was measured during transitions from dark to 130 μmol photons m^‐2^ s^‐1^ of green actinic light. Absorbance difference between 546 and 520 nm (electrochromic shift, ECS) was measured with a JTS‐150 spectrometer, and light‐induced *pmf* was determined from the dark interval relaxation kinetics of the ECS signal. Conductivity of the thylakoid membrane (gH^+^) after 88 s or 60 s of illumination was determined as the inverse of the time constant of first‐order post‐illumination decay kinetics of the ECS signal. Thylakoid proton flux (vH^+^), calculated as *pm*f × gH^+^. ECS measurements were done both before the heat period and during the heat period to distinguish acute short‐term effects of heat treatment from long‐term acclimatory responses. Statistical significance according to Student's t‐test is indicated with an asterisk (* = < 0.05, ** = < 0.01, *** = < 0.001). (*n* = 6 individual plants). Standard deviations are indicated with error bars.

To analyze the effects of downregulated Cyt *b*_*6*_ *f* and ATP synthase on proton motive force (*pmf*), electrochromic shift (ECS) was monitored during dark to light transition (Figure 5; Supporting Information Figures S3 and S4). In order to distinguish short‐term heat‐induced differences from the long‐term acclimatory responses resulting from decreased Cyt *b*_*6*_ *f* levels, measurements for heat‐acclimated plants were done before the onset of heat period (Figure 5e; Supporting Information Figure S3a–d) as well as at mid‐day in the middle of the heat period (Figure 5f; Supporting Information Figure S3e–g), whereas control plants were kept in constant 23°C. In concordance with the reduced Cyt *b*_*6*_ *f* levels, heat‐acclimated plants showed decreased *pmf* compared to Ctrl plants (Figure 5e; Supporting Information Figure S3a) when measurements were conducted before the onset of heat period. There were no major differences in thylakoid membrane conductivity (gH^+^) between Ctrl and HS plants, but proton flux to lumen (vH^+^) was significantly reduced in HS plants (Figure 5e; Supporting Information Figure S3c,d) in agreement with the decreased PSII and PSI electron transport rates. Interestingly, when measured in the middle of the heat period, the heat‐acclimated plants showed higher *pmf*, and significantly higher vH^+^ compared to Ctrl plants (Figure 5f; Supporting Information Figure S3e–g). Further, during the heat period a slightly higher proportion of Cyt *f* remained oxidized during dark‐to‐light transitions in the heat‐acclimated plants in comparison to Ctrl plants (Supporting Information Figure S4a), while the reduction rates of Cyt *f* were similar between treatments (Supporting Information Figure S4b). Earlier reports have suggested CET induction and thus lumen protonation upon exposure to high temperatures and therefore we tested, whether the accumulation of CET‐related proteins NDHL or PGR5 are altered in heat acclimated plants. There were no differences in the level of PGR5 (Supporting Information Figure S5), but the NdhL subunit of the CET‐related PSI‐NDH complex (Peng and Shikanai 2011) was significantly upregulated in the heat‐acclimated plants compared to Ctrl plants (Supporting Information Figure S5).

### 3.5 Heat‐Acclimated Plants Show Reduced Carbon Assimilation Rates

Since high temperatures have been shown to inhibit photosynthesis by decreasing Rubisco activity via inhibiting RCA (Crafts‐Brandner and Salvucci 2000; Salvucci et al. 2001) and increasing photorespiration, we assessed carbon assimilation capacity of heat‐acclimated plants (Figure 6a). Net carbon assimilation rates as well as stomatal conductance were significantly reduced in heat‐acclimated plants compared to Ctrl plants (Figure 6). However, since the chlorophyll content was reduced in the heat‐acclimated plants and the carbon assimilation rates were not normalized to the fraction of light absorbed, the apparent reduction in carbon assimilation may be overestimated. Interestingly, there were no significant differences in total RCA levels between Ctrl and HS plants, but in accordance with the previous findings (Rokka et al. 2001), more RCA was associated with the thylakoid membranes in HS plants (Supporting Information Figure S6). No differences were observed in the total amount of Rubisco or in the amount of thylakoid membrane‐associated Rubisco between Ctrl and HS plants (Supporting Information Figure S6).

**Figure 6:**
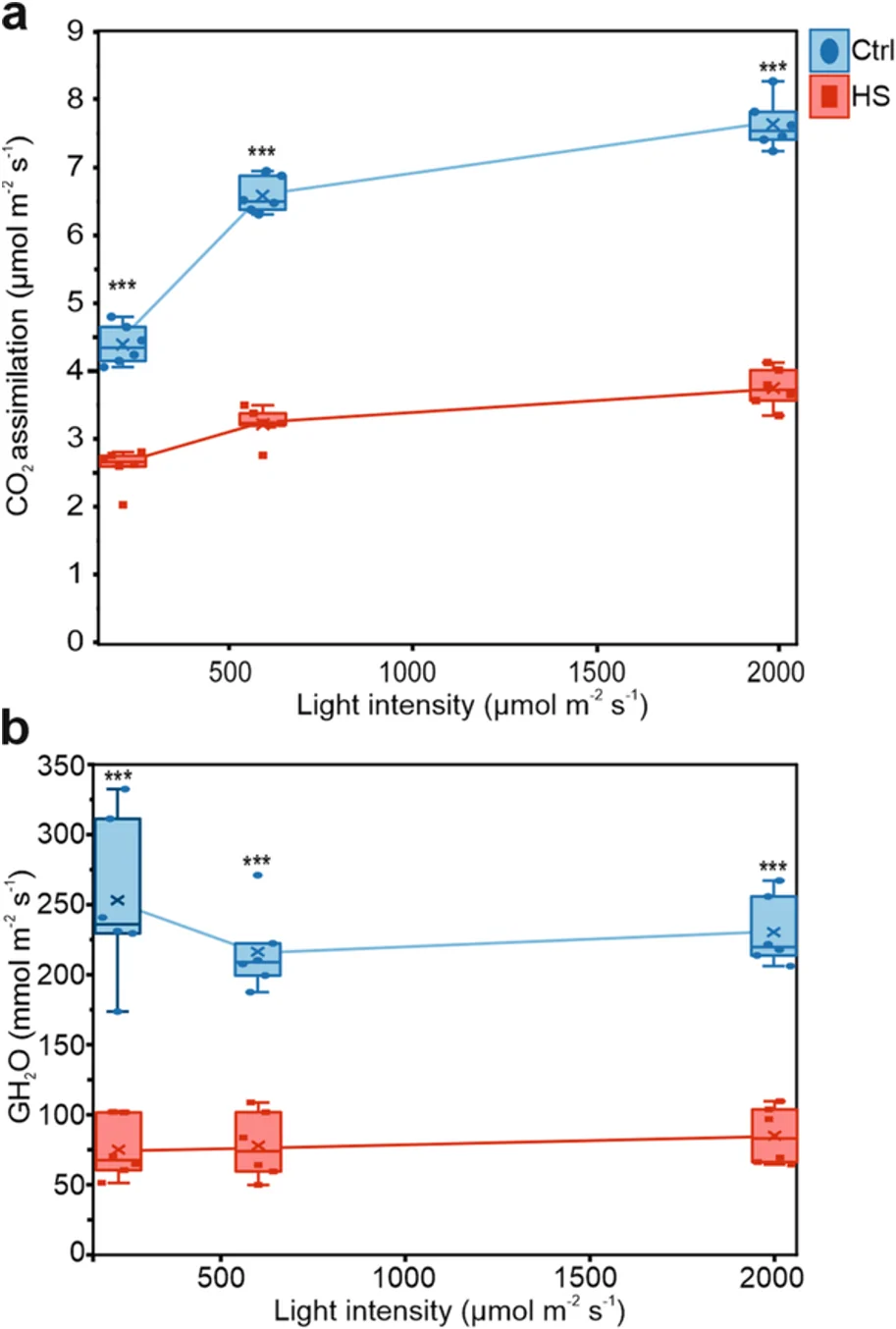
The effect of daily heat stress (HS) on net carbon assimilation and stomata conductivity. a) Net carbon assimilation rate (A_n_) and b) leaf conductance to water vapor were analyzed from control (Ctrl) and heat‐acclimated (HS) plants with GFS‐3000 (Walz). Measurements were done at mid‐day in the middle of heat period using three different light intensities (200, 600, and 2000 μmol m^−2^ s^−1^) and illuminating leaf for 5 min with each intensity at 20°C. Statistical significance according to Student's t‐test is indicated with an asterisk (* = < 0.05, ** = < 0.01, *** = < 0.001). (*n* = 6 individual plants). Standard deviations are indicated with error bars.

## 4 Discussion

Increase in ambient temperature affects multiple aspects of plant metabolism, especially photosynthetic reactions (Berry and Bjorkman 1980). Currently, holistic understanding of the effects of physiologically relevant long‐term heat stress on the thylakoid membrane reactions is completely lacking. Long‐term changes in environmental conditions trigger changes in the consumption of ATP and NADPH, and the stoichiometry of photosynthetic complexes is dynamically regulated to meet the metabolic needs of the plant (Schöttler and Tóth 2014). It has been suggested that these dynamic changes are mediated by the redox state of photosynthetic electron transfer chain, that is, PQ pool and Cyt *b*_*6*_ *f*, which act as a signaling network to regulate gene expression levels in nucleus and chloroplast (Anderson et al. 1997; Pfannschmidt 2003).

Long‐term daily heat stress leads to a pronounced reduction in chlorophyll (Chl) content (Figure 1c), consistent with previous findings (Fatma et al. 2021; Mustafa et al. 2021). This decrease has been attributed to enhanced accumulation of chlorophyllase and Chl‐degrading peroxidases in plants (Rossi et al. 2017). Previous studies have shown almost complete destacking of the thylakoid membrane upon exposure to high temperature (Gounaris et al. 1983, 1984). In heat‐acclimated plants, although the grana stacks are slightly reduced in height, the number of grana per chloroplast is increased (Figure 2a‐c). Moreover, heat‐acclimated plants exhibit higher frequency of C2S2M2 and C2S2M forms of PSII‐LHCII supercomplexes compared to Ctrl plants (Figure 3a). The higher accumulation of the large forms of PSII‐LHCII likely reflects a physiologically relevant enhancement of the interaction of LHCII with PSII. This would imply that PSII‐LHCII is rather stable under long‐term heat stress, which is in striking contrast with earlier reports demonstrating D1 degradation (Komayama et al. 2007; Yamashita et al. 2008) and complete physical disconnection of the LHCII antenna from the PSII core upon heat stress (Armond et al. 1980; Sundby et al. 1986; Srivastava et al. 1997; Mathur et al. 2011). It is also possible that changes in thylakoid membrane lipid composition affects the stability of the PSII‐LHCII complexes against detergent treatment.

Strikingly, we show that electron flow beyond Q_A_ is suppressed by dramatic Cyt *b*_*6*_ *f* downregulation (Figure 4a; Figure 5d), which explains the observed reduction in PSII photochemistry, ∆F/F_m_’ and rETRII, (Figure 3a‐d). Decrease in the Cyt *b*_*6*_ *f* content leads to donor side limitation of PSI and more oxidized PC and P700 (Figure 5a‐c), which likely serves to protect PSI from excessive reduction and ROS generation. Protection of PSI is crucial, as it lacks similar repair machinery as PSII: once PSI is damaged, the repair process requires *de novo* synthesis and assembly of the entire complex (Sonoike 2011). In agreement with the lower Cyt *b*_*6*_ *f* content, we observed reduced *pmf* in heat‐acclimated plants compared to Ctrl plants (Figure 5e) when measurements were taken prior to the daily heat treatment. Interestingly, despite the lower ATP synthase content in heat‐acclimated plants (Figure 4a; Figure 5d), gH^+^ levels remained similar between the two groups (Figure 5e; Supporting Information Figure S3c). This observation is consistent with findings by Rott et al. (2011), who reported that ATP synthase content can be reduced by up to 50% without affecting gH^+^. Notably, when *pmf* was measured during the heat period (Figure 5f), proton flux to the lumen was actually higher in heat‐acclimated plants (Supporting Information Figure S3g). This could indicate increased CET that is activated during the heat period. Indeed, previous studies have reported activation of CET under high temperature (Bukhov et al. 1999; Schrader et al. 2004; Wang et al. 2006; Zhang and Sharkey 2009; Zhang et al. 2023), which has been hypothesized to maintain sufficient ATP content upon heat‐induced thylakoid leakiness. We did observe higher accumulation of NdhL protein, which represents CET‐related PSI‐NDH complex (Peng and Shikanai 2011) in the heat‐acclimated plants compared to Ctrl (Figure 5f; Supporting Information Figures S4a and S5). During the heat period, higher proportion of oxidized Cyt *f* was observed in the heat‐acclimated plants in comparison to control plants (Supporting Information Figure S4a). More oxidized Cyt *f* could reflect activation of photosynthetic control due to increased *pmf*. However, the possible involvement of CET and/or other alternative electron transfer pathways upon heat exposure require more research.

Taken together, our results demonstrate that the mechanisms limiting photosynthesis under single heat exposure are very different from those activated upon long‐term repeated exposures, applied in our experimental setup. Contrasting to earlier studies that demonstrate PSII‐LHCII as being the most sensitive component of ETC under heat (Komayama et al. 2007; Yamashita et al. 2008; Armond et al. 1980; Sundby et al. 1986; Srivastava et al. 1997; Mathur et al. 2011), we show that under prolonged physiologically relevant heat stress, PSII‐LHCII remain structurally intact (Figure 3a,b), and the reduced PSII photochemistry is mostly explained by blockages of the electron transfer downstream of Q_A_. Indeed, we show that under long‐term heat stress, Cyt *b*_*6*_ *f* is dramatically downregulated (Figure 5d) leading to restricted electron flow from PQ pool to PSI and thus more oxidized P700 (Figure 5a–c). We propose that the reduced Cyt *b*_*6*_ *f* content represents an acclimatory mechanism that protect PSI complex from over‐reduction in conditions, where downstream metabolism is inhibited (Figures 6a, b and 7). In accordance with earlier reports, heat stress affects thylakoid ultrastructure, but unlike sudden extreme heat exposure, long‐term heat stress does not result in complete destacking of grana (Gounaris et al. 1983, 1984), but only to mild decrease in grana height (Figure 2a–c).

**Figure 7:**
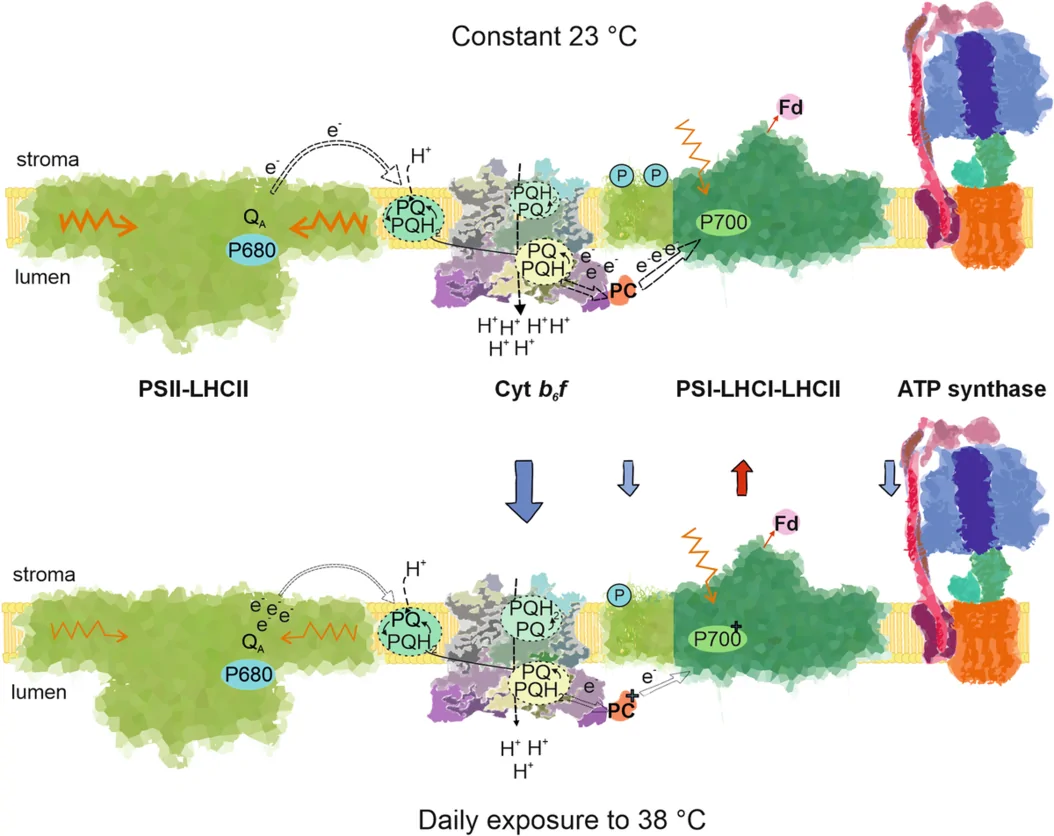
Summary of the effects of long‐term daily heat exposure to photosynthetic light reactions. Compared to control conditions (constant 23°C), plants exposed daily to 38°C for 4 h, exhibit pronounced changes in the structure and function of the photosynthetic machinery. Blue/red arrows on top of complexes indicate down‐/upregulation of the complexes. Despite retaining structurally intact PSII–LHCII supercomplexes, heat‐acclimated plants show reduced PSII photochemical efficiency. Downregulation of Cyt *b*_*6*_ *f* decreases electron flux to PSI, leading to more oxidized plastocyanin (PC) and P700.

## Supporting information


Supporting File 1

Supporting File 2

Supporting File 3

## Data Availability

The data that supports the findings of this study are available in the supporting information of this article.

## References

[pce70730-bib-0001] Allakhverdiev, S. I. , V. D. Kreslavski , V. V. Klimov , D. A. Los , R. Carpentier , and P. Mohanty . 2008. “Heat Stress: An Overview of Molecular Responses in Photosynthesis.” Photosynthesis Research 98: 541–550. 10.1007/s11120-008-9331-0. 18649006

[pce70730-bib-0002] Anderson, J. M. , G. Dean Price , W. Soon Chow , A. B. Hope , and M. R. Badger . 1997. “Reduced Levels of Cytochrome *bf* Complex in Transgenic Tobacco Leads to Marked Photochemical Reduction of the Plastoquinone Pool, Without Significant Change in Acclimation to Irradiance.” Photosynthesis Research 53: 215–227. 10.1023/A:1005856615915.

[pce70730-bib-0003] Armond, P. A. , O. Björkman , and L. A. Staehelin . 1980. “Dissociation of Supramolecular Complexes in Chloroplast Membranes A Manifestation of Heat Damage to the Photosynthetic Apparatus.” Biochimica et Biophysica Acta (BBA) ‐ Biomembranes 601: 433–442. 10.1016/0005-2736(80)90547-7. 7417433

[pce70730-bib-0005] Avenson, T. J. , J. A. Cruz , A. Kanazawa , and D. M. Kramer . 2005. “Regulating the Proton Budget of Higher Plant Photosynthesis.” Proceedings of the National Academy of Sciences 102: 9709–9713. 10.1073/pnas.0503952102. PMC1172270 15972806

[pce70730-bib-0007] Berry, J. , and O. Bjorkman . 1980. “Photosynthetic Response and Adaptation to Temperature in Higher Plants.” Annual Review of Plant Physiology 31: 491–543. 10.1146/annurev.pp.31.060180.002423.

[pce70730-bib-0008] Bukhov, N. G. , C. Wiese , S. Neimanis , and U. Heber . 1999. “Heat Sensitivity of Chloroplasts and Leaves: Leakage of Protons From Thylakoids and Reversible Activation of Cyclic Electron Transport.” Photosynthesis Research 59: 81–93. 10.1023/A:1006149317411.

[pce70730-bib-0009] Crafts‐Brandner, S. J. , and M. E. Salvucci . 2000. “Rubisco Activase Constrains the Photosynthetic Potential of Leaves at High Temperature and Co 2.” Proceedings of the National Academy of Sciences 97: 13430–13435. 10.1073/pnas.230451497. PMC27241 11069297

[pce70730-bib-0010] Cruz, J. A. 2004. “Plasticity in Light Reactions of Photosynthesis for Energy Production and Photoprotection.” Journal of Experimental Botany 56: 395–406. 10.1093/jxb/eri022. 15533877

[pce70730-bib-0011] Demmig‐Adams, B. 1990. “Carotenoids and Photoprotection in Plants: A Role for the Xanthophyll Zeaxanthin.” Biochimica et Biophysica Acta (BBA) ‐ Bioenergetics 1020: 1–24.

[pce70730-bib-0012] Enami, I. , M. Kitamura , T. Tomo , Y. Isokawa , H. Ohta , and S. Katoh . 1994. “Is the Primary Cause of Thermal Inactivation of Oxygen Evolution in Spinach PS II Membranes Release of the Extrinsic 33 kDa Protein or of Mn?” Biochimica et Biophysica Acta (BBA) ‐ Bioenergetics 1186: 52–58. 10.1016/0005-2728(94)90134-1.

[pce70730-bib-0013] Fatma, M. , N. Iqbal , Z. Sehar , et al. 2021. “Methyl Jasmonate Protects the PS II System by Maintaining the Stability of Chloroplast D1 Protein and Accelerating Enzymatic Antioxidants in Heat‐Stressed Wheat Plants.” Antioxidants 10: 1216. 10.3390/antiox10081216. PMC8388886 34439464

[pce70730-bib-0014] Feller, U. , S. J. Crafts‐Brandner , and M. E. Salvucci . 1998. “Moderately High Temperatures Inhibit Ribulose‐1,5‐Bisphosphate Carboxylase/Oxygenase (Rubisco) Activase‐Mediated Activation of Rubisco1.” Plant Physiology 116: 539–546. 10.1104/pp.116.2.539. PMC35111 9490757

[pce70730-bib-0015] Genty, B. , J. M. Briantais , and N. R. Baker . 1989. “The Relationship Between the Quantum Yield of Photosynthetic Electron Transport and Quenching of Chlorophyll Fluorescence.” Biochimica et Biophysica Acta (BBA) ‐ General Subjects 990: 87–92.

[pce70730-bib-0016] Gounaris, K. , A. P. R. Brain , P. J. Quinn , and W. P. Williams . 1983. “Structural and Functional Changes Associated With Heat‐Induced Phase‐Separations of Non‐Bilayer Lipids in Chloroplast Thylakoid Membranes.” FEBS Letters 153: 47–52. 10.1016/0014-5793(83)80117-3.

[pce70730-bib-0017] Gounaris, K. , A. R. R. Brain , P. J. Quinn , and W. P. Williams . 1984. “Structural Reorganisation of Chloroplast Thylakoid Membranes in Response to Heat‐Stress.” Biochimica et Biophysica Acta (BBA) ‐ Bioenergetics 766: 198–208. 10.1016/0005-2728(84)90232-9.

[pce70730-bib-0018] Havaux, M. 1993. “Characterization of Thermal Damage to the Photosynthetic Electron Transport System in Potato Leaves.” Plant Science 94: 19–33. 10.1016/0168-9452(93)90003-I.

[pce70730-bib-0019] Hyman, S. , and R. P. Jarvis . 2011. “Studying Arabidopsis Chloroplast Structural Organisation Using Transmission Electron Microscopy.” Methods in Molecular Biology 774: 113–132. 10.1007/978-1-61779-234-2_8. 21822836

[pce70730-bib-0020] Inoué, H. , T. Kitamura , and M. Noguchi . 1987. “Heat Inactivation of Electron Transport Reactions in Photosystem II Particles.” Physiologia Plantarum 71: 441–447. 10.1111/j.1399-3054.1987.tb02881.x.

[pce70730-bib-0021] Intergovernmental Panel on Climate Change (IPCC) . 2023. Climate Change 2021 – The Physical Science Basis: Working Group I Contribution to the Sixth Assessment Report of the Intergovernmental Panel on Climate Change. Cambridge University Press. 10.1017/9781009157896.

[pce70730-bib-0022] Ivanov, A. G. , and M. Y. Velitchkova . 1990. “Heat Induced Changes in the Efficiency of P700 Photo‐Oxidation in Pea Chloroplast Membranes.” Journal of Photochemistry and Photobiology, B: Biology 4: 307–320. 10.1016/1011-1344(90)85036-V.

[pce70730-bib-0023] Ivanov, A. G. , M. Y. Velitchkova , S. I. Allakhverdiev , and N. P. A. Huner . 2017. “Heat Stress‐Induced Effects of Photosystem I: An Overview of Structural and Functional Responses.” Photosynthesis Research 133: 17–30. 10.1007/s11120-017-0383-x. 28391379

[pce70730-bib-0024] Järvi, S. , M. Suorsa , V. Paakkarinen , and E. M. Aro . 2011. “Optimized Native Gel Systems for Separation of Thylakoid Protein Complexes: Novel Super‐ and Mega‐Complexes.” Biochemical Journal 439: 207–214. 10.1042/BJ20102155. 21707535

[pce70730-bib-0074] Kettunen, R. , E. Tyystjärvi , and E. M. Aro . 1996. “Degradation Pattern of Photosystem II Reaction Center Protein D1 in Intact Leaves. The Major Photoinhibition‐Induced Cleavage Site in D1 Polypeptide Is Located Amino Terminally of the De Loop.” Plant Physiology 111: 1183–1190. http://www.jstor.org/stable/4277272. 10.1104/pp.111.4.1183 PMC160995 8756500

[pce70730-bib-0025] Klughammer, C. , and U. Schreiber . 2016. “Deconvolution of Ferredoxin, Plastocyanin, and P700 Transmittance Changes in Intact Leaves With a New Type of Kinetic LED Array Spectrophotometer.” Photosynthesis Research 128: 195–214. 10.1007/s11120-016-0219-0. PMC4826414 26837213

[pce70730-bib-0027] Komayama, K. , M. Khatoon , D. Takenaka , et al. 2007. “Quality Control of Photosystem Ii: Cleavage and Aggregation of Heat‐Damaged D1 Protein in Spinach Thylakoids.” Biochimica et Biophysica Acta (BBA) ‐ Bioenergetics 1767: 838–846. 10.1016/j.bbabio.2007.05.001. 17543883

[pce70730-bib-0029] Kramer, D. M. , and C. A. Sacksteder . 1998. “A Diffused‐Optics Flash Kinetic Spectrophotometer (DOFS) for Measurements of Absorbance Changes in Intact Plants in the Steady‐State.” Photosynthesis Research 56: 103–112. 10.1023/A:1005968211506. 16228356

[pce70730-bib-0028] Kramer, D. M. , G. Johnson , O. Kiirats , and G. E. Edwards . 2004. “New Fluorescence Parameters for the Determination of QA Redox State and Excitation Energy Fluxes.” Photosynthesis Research 79: 209–218. 10.1023/B:PRES.0000015391.99477.0d 16228395

[pce70730-bib-0073] Laemmli, U. K. 1970. “Cleavage of Structural Proteins During the Assembly of the Head of Bacteriophage T4.” Nature 227: 680–685. 10.1038/227680a0. 5432063

[pce70730-bib-0030] Law, R. D. , and S. J. Crafts‐Brandner . 1999. “Inhibition and Acclimation of Photosynthesis to Heat Stress Is Closely Correlated With Activation of Ribulose‐1,5‐bisphosphate Carboxylase/Oxygenase.” Plant Physiology 120: 173–182. 10.1104/pp.120.1.173. PMC59249 10318695

[pce70730-bib-0031] Mathur, S. , A. Jajoo , P. Mehta , and S. Bharti . 2011. “Analysis of Elevated Temperature‐Induced Inhibition of Photosystem Ii Using Chlorophyll a Fluorescence Induction Kinetics in Wheat Leaves (*Triticum aestivum*).” Plant Biology 13: 1–6. 10.1111/j.1438-8677.2009.00319.x. 21143718

[pce70730-bib-0032] Mohanty, P. , B. Vani , and J. S. S. Prakash . 2002. “Elevated Temperature Treatment Induced Alteration in Thylakoid Membrane Organization and Energy Distribution Between the Two Photosystems in *Pisum sativum* .” Zeitschrift für Naturforschung C 57: 836–842. 10.1515/znc-2002-9-1014. 12440721

[pce70730-bib-0033] Moore, C. E. , K. Meacham‐Hensold , P. Lemonnier , et al. 2021. “The Effect of Increasing Temperature on Crop Photosynthesis: From Enzymes to Ecosystems.” Journal of Experimental Botany 72: 2822–2844. 10.1093/jxb/erab090. PMC8023210 33619527

[pce70730-bib-0034] Mustafa, T. , A. Sattar , A. Sher , et al. 2021. “Exogenous Application of Silicon Improves the Performance of Wheat Under Terminal Heat Stress by Triggering Physio‐Biochemical Mechanisms.” Scientific Reports 11: 23170. 10.1038/s41598-021-02594-4. PMC8633343 34848766

[pce70730-bib-0035] Nellaepalli, S. , N. R. Mekala , O. Zsiros , P. Mohanty , and R. Subramanyam . 2011. “Moderate Heat Stress Induces State Transitions in *Arabidopsis thaliana* .” Biochimica et Biophysica Acta (BBA) ‐ Bioenergetics 1807: 1177–1184. 10.1016/j.bbabio.2011.05.016. 21640068

[pce70730-bib-0036] Oxborough, K. , and N. R. Baker . 1997. “Resolving Chlorophyll a Fluorescence Images of Photosynthetic Efficiency into Photochemical and Non‐Photochemical Components – Calculation of qP and Fv’/Fm’ Without Measuring Fo.” Photosynthesis Research 54: 135–142.

[pce70730-bib-0037] Peng, L. , and T. Shikanai . 2011. “Supercomplex Formation With Photosystem I is Required for the Stabilization of the Chloroplast NADH Dehydrogenase‐Like Complex in Arabidopsis.” Plant Physiology 155: 1629–1639. 10.1104/pp.110.171264. PMC3091109 21278308

[pce70730-bib-0038] Pfannschmidt, T. 2003. “Chloroplast Redox Signals: How Photosynthesis Controls its Own Genes.” Trends in Plant Science 8: 33–41. 10.1016/s1360-1385(02)00005-5. 12523998

[pce70730-bib-0039] Porra, R. J. , W. A. Thompson , and P. E. Kriedemann . 1989. “Determination of Accurate Extinction Coefficients and Simultaneous Equations for Assaying Chlorophylls A and B Extracted With Four Different Solvents: Verification of the Concentration of Chlorophyll Standards by Atomic Absorption Spectroscopy.” Biochimica et Biophysica Acta (BBA) ‐ Bioenergetics 975: 384–394. 10.1016/S0005-2728(89)80347-0.

[pce70730-bib-0040] Pospíšil, P. , J. Skotnica , and J. Nauš . 1998. “Low and High Temperature Dependence of Minimum F0 and Maximum FM Chlorophyll Fluorescence In Vivo.” Biochimica et Biophysica Acta (BBA) ‐ Bioenergetics 1363: 95–99. 10.1016/S0005-2728(97)00095-9. 9507075

[pce70730-bib-0041] Pshybytko, N. L. , J. Kruk , L. F. Kabashnikova , and K. Strzalka . 2008. “Function of Plastoquinone in Heat Stress Reactions of Plants.” Biochimica et Biophysica Acta (BBA) ‐ Bioenergetics 1777: 1393–1399. 10.1016/j.bbabio.2008.08.005. 18778684

[pce70730-bib-0042] Rantala, M. , V. Paakkarinen , and E. M. Aro . 2018. “Analysis of Thylakoid Membrane Protein Complexes by Blue Native Gel Electrophoresis.” Journal of Visualized Experiments: JoVE 139: 58369. 10.3791/58369. PMC6235366 30320749

[pce70730-bib-0047] Rokka, A. , L. Zhang , and E. M. Aro . 2001. “Rubisco Activase: An Enzyme With a Temperature‐Dependent Dual Function?” The Plant Journal 25: 463–471. 10.1046/j.1365-313x.2001.00981.x. 11260502

[pce70730-bib-0048] Rossi, S. , P. Burgess , D. Jespersen , and B. Huang . 2017. “Heat‐Induced Leaf Senescence Associated With Chlorophyll Metabolism in Bentgrass Lines Differing in Heat Tolerance.” Crop Science 57: 169–178. 10.2135/cropsci2016.06.0542.

[pce70730-bib-0075] Rott, M. , N. F. Martins , W. Thiele , et al. 2011. “ATP Synthase Repression in Tobacco Restricts Photosynthetic Electron Transport, CO2 Assimilation, and Plant Growth by Overacidification of the Thylakoid Lumen.” Plant Cell 23: 304–321. 10.1105/tpc.110.079111. PMC3051256 21278125

[pce70730-bib-0049] Salvucci, M. E. , K. W. Osteryoung , S. J. Crafts‐Brandner , and E. Vierling . 2001. “Exceptional Sensitivity of Rubisco Activase to Thermal Denaturation In Vitro and In Vivo.” Plant Physiology 127: 1053–1064. PMC129275 11706186

[pce70730-bib-0051] Santarius, K. A. 1976. “Sites of Heat Sensitivity in Chloroplasts and Differential Inactivation of Cyclic and Noncyclic Photophosphorylation by Heating.” Journal of Thermal Biology 1: 101–107. 10.1016/0306-4565(76)90028-0.

[pce70730-bib-0053] Schöttler, M. A. , and S. Z. Tóth . 2014. “Photosynthetic Complex Stoichiometry Dynamics in Higher Plants: Environmental Acclimation and Photosynthetic Flux Control.” Frontiers in Plant Science 5: 188. 10.3389/fpls.2014.00188. PMC4026699 24860580

[pce70730-bib-0054] Schrader, S. M. , R. R. Wise , W. F. Wacholtz , D. R. Ort , and T. D. Sharkey . 2004. “Thylakoid Membrane Responses to Moderately High Leaf Temperature in Pima Cotton.” Plant, Cell and Environment 27: 725–735. 10.1111/j.1365-3040.2004.01172.x.

[pce70730-bib-0055] Semenova, G. A. 2004. “Structural Reorganization of Thylakoid Systems in Response to Heat Treatment.” Photosynthetica 42: 521–527. 10.1007/S11099-005-0008-z.

[pce70730-bib-0056] Sharkey, T. D. 2005. “Effects of Moderate Heat Stress on Photosynthesis: Importance of Thylakoid Reactions, Rubisco Deactivation, Reactive Oxygen Species, and Thermotolerance Provided by Isoprene.” Plant, Cell and Environment 28: 269–277. 10.1111/j.1365-3040.2005.01324.x.

[pce70730-bib-0057] Sharkey, T. D. , and R. Zhang . 2010. “High Temperature Effects on Electron and Proton Circuits of Photosynthesis.” Journal of Integrative Plant Biology 52: 712–722. 10.1111/j.1744-7909.2010.00975.x. 20666927

[pce70730-bib-0058] Shimizu, H. , L. Peng , F. Myouga , R. Motohashi , K. Shinozaki , and T. Shikanai . 2008. “CRR23/NdhL is a Subunit of the Chloroplast NAD(P)H Dehydrogenase Complex in Arabidopsis.” Plant and Cell Physiology 49: 835–842. 10.1093/pcp/pcn058. 18388109

[pce70730-bib-0059] Sonoike, K. 2011. “Photoinhibition of Photosystem I.” Physiologia Plantarum 142: 56–64. 10.1111/j.1399-3054.2010.01437.x. 21128947

[pce70730-bib-0060] Srivastava, A. , B. Guissé , H. Greppin , and R. J. Strasser . 1997. “Regulation of Antenna Structure and Electron Transport in Photosystem II of Pisum Sativum under Elevated Temperature Probed by the Fast Polyphasic Chlorophyll a Fluorescence Transient: Okjip.” Biochimica et Biophysica Acta (BBA) ‐ Bioenergetics 1320: 95–106. 10.1016/S0005-2728(97)00017-0.

[pce70730-bib-0061] Sundby, C. , A. Melis , P. Mäenpää , and B. Andersson . 1986. “Temperature‐Dependent Changes in the Antenna Size of Photosystem II. Reversible Conversion of Photosystem IIα to Photosystem IIβ.” Biochimica et Biophysica Acta (BBA) ‐ Bioenergetics 851: 475–483. 10.1016/0005-2728(86)90084-8.

[pce70730-bib-0062] Wang, P. , W. Duan , A. Takabayashi , et al. 2006. “Chloroplastic NAD(P)H Dehydrogenase in Tobacco Leaves Functions in Alleviation of Oxidative Damage Caused by Temperature Stress.” Plant Physiology 141: 465–474. 10.1104/pp.105.070490. PMC1475475 16428601

[pce70730-bib-0063] Wellburn, A. R. 1994. “The Spectral Determination of Chlorophylls A and B, as Well as Total Carotenoids, Using Various Solvents With Spectrophotometers of Different Resolution.” Journal of Plant Physiology 144: 307–313. 10.1016/S0176-1617(11)81192-2.

[pce70730-bib-0064] World Meteorological Organization . (2025). WMO Global Annual to Decadal Climate Update 2025‐2029.

[pce70730-bib-0066] Yamashita, A. , N. Nijo , P. Pospíšil , et al. 2008. “Quality Control of Photosystem II.” Journal of Biological Chemistry 283: 28380–28391. 10.1074/jbc.M710465200. PMC2661399 18664569

[pce70730-bib-0067] Zhang, R. , and T. D. Sharkey . 2009. “Photosynthetic Electron Transport and Proton Flux Under Moderate Heat Stress.” Photosynthesis Research 100: 29–43. 10.1007/s11120-009-9420-8. 19343531

[pce70730-bib-0068] Zhang, Y. , Y. Fan , X. Lv , X. Zeng , Q. Zhang , and P. Wang . 2023. “Deficiency in NDH‐Cyclic Electron Transport Retards Heat Acclimation of Photosynthesis in Tobacco over Day and Night Shift.” Frontiers in Plant Science 14: 1267191. 10.3389/fpls.2023.1267191. PMC10644794 38023894

[pce70730-bib-0069] Zhao, C. , B. Liu , S. Piao , et al. 2017. “Temperature Increase Reduces Global Yields of Major Crops in Four Independent Estimates.” Proceedings of the National Academy of Sciences 114: 9326–9331. 10.1073/pnas.1701762114. PMC5584412 28811375

